# HPLC–HRMS/MS Characterization, Molecular Docking, and Comparative Genotoxicity of Free and Liposome-Encapsulated *Aesculus hippocastanum* Seed Extract

**DOI:** 10.3390/ijms27188372

**Published:** 2026-09-20

**Authors:** Boyka Andonova-Lilova, Plamena Staleva, Zhanina Petkova, Emilio Mateev, Tzvetelina Zagorcheva, Viktoria Milkova, Anna Gyurova, Petar Martinov, Viktor Rashev, Neli Vilhelmova-Ilieva

**Affiliations:** 1 Research and Development and Innovation Consortium, 1784 Sofia, Bulgaria; boika_andonova@hotmail.com (B.A.-L.); tzvetelina.zagorcheva@gmail.com (T.Z.); 2 Department Agrobiotechnology, Agricultural Academy, AgroBio Institute, 8 Dragan Tzankov Blvd., 1164 Sofia, Bulgaria; 3 Laboratory Organic Synthesis and Stereochemistry, Institute of Organic Chemistry with Centre of Phytochemistry, Bulgarian Academy of Sciences, 1113 Sofia, Bulgaria; zhanina.petkova@orgchm.bas.bg; 4 Centre of Competence “Sustainable Utilization of Bio-Resources and Waste of Medicinal and Aromatic Plants for Innovative Bioactive Products” (BIORESOURCES BG), 1000 Sofia, Bulgaria; 5 Department of Pharmaceutical Chemistry, Faculty of Pharmacy, Medical University, 1000 Sofia, Bulgaria; e.mateev@pharmfac.mu-sofia.bg; 6 Institute of Physical Chemistry ‘Acad. R. Kaischew’, Bulgarian Academy of Sciences, 1113 Sofia, Bulgaria; vmilkova@ipc.bas.bg (V.M.); any_gyurova@abv.bg (A.G.); petar.martinov96@gmail.com (P.M.); 7 Stephan Angeloff Institute of Microbiology, Bulgarian Academy of Sciences, Acad. G. Bonchev Str., 26, 1113 Sofia, Bulgaria; vpr2012@abv.bg

**Keywords:** *Aesculus hippocastanum*, chemical profiling, HPLC–HRMS/MS, comet assay, molecular docking, hesperidin, quercetin, Nsp5, Nsp12, coronavirus

## Abstract

Plant extracts remain an important source of antiviral agents, while nanocarrier-based delivery systems have emerged as a promising strategy for improving their therapeutic safety and efficacy. Building on previous findings indicating that nanoliposomal encapsulation improved the anti-coronavirus activity of *Aesculus hippocastanum* seed extract against human coronavirus strain OC43 (HCoV-OC43), the present study evaluated whether encapsulation reduces the genotoxic effects of the extract and explored a potential molecular basis for the previously observed antiviral activity. Genotoxicity assessment using the alkaline comet assay in MRC-5 human lung fibroblasts demonstrated that at 1 mg/mL, liposomal encapsulation was associated with an approximately 86.0% reduction in %Tail DNA, from 21.301 ± 0.368% for the free extract to 2.984 ± 1.523% for the liposome-encapsulated extract. In parallel, chemical profiling by high-performance liquid chromatography coupled with high-resolution tandem mass spectrometry (HPLC–HRMS/MS) enabled the identification and tentative annotation of 46 metabolites, including triterpenoid saponins, flavonoids, indole-3-acetic acid glycosides, and organic acids. Based on this phytochemical profile, representative constituents were subjected to molecular docking against the severe acute respiratory syndrome coronavirus 2 (SARS-CoV-2) nonstructural proteins Nsp5 and Nsp12 to investigate their potential contribution to the previously demonstrated antiviral activity of the extract. Among the evaluated compounds, hesperidin exhibited the most favorable predicted binding affinity toward both targets, while quercetin also showed favorable interactions, suggesting that flavonoid constituents may contribute to the antiviral activity of the extract. These findings indicate that liposomal encapsulation improves the biological safety profile of *A. hippocastanum* seed extract, while phytochemical characterization and molecular docking provide preliminary insights into the constituents that may contribute to its previously demonstrated anti-coronavirus activity.

## 1. Introduction

Human coronaviruses are a significant group of respiratory pathogens characterized by a distinctive and well-studied virion morphology. The virions exhibit significant pleomorphism, with heterogeneous distribution of the S and M proteins within individual particles [1,2,3]. As enveloped viruses, they also possess a bilayer lipid envelope derived from intracellular membranes during virus budding [2,4]. The Coronaviridae family is classified into four monophyletic subgenera within the subfamily *Orthocoronavirinae*: Alphacoronavirus, Betacoronavirus, Deltacoronavirus, and Gammacoronavirus. The current classification recognizes 39 species, reflecting extensive analyses of over 2000 viral genomes [5]. The seven known human coronaviruses include four common strains (229E, OC43, NL63, and HKU1) that typically cause mild respiratory illness, and three highly pathogenic strains (SARS, MERS, and SARS-2) associated with severe respiratory illness. These viruses exhibit different cytopathic effects, cell tropism, and immune interactions that contribute to their diverse clinical manifestations [6].

The availability of specific antiviral treatments for coronaviruses in the SARS and MERS outbreaks was limited, and existing therapies were mainly focused on providing supportive care. IFNs have shown only partial efficacy against coronaviruses. The combination of IFN-α with other antiviral agents, such as remdesivir, has shown synergistic effects against viruses such as SARS-2, suggesting the potential for broader use of IFN-α-based combination therapies [7]. The SARS and MERS outbreaks prompted extensive research on these viruses, leading to the identification of multiple potential targets for antiviral drugs, including viral proteases, polymerases, and entry proteins [8].

Remdesivir, marketed as Veklury, is a broad-spectrum antiviral drug originally developed for the treatment of hepatitis C and later tested for Ebola and Marburg infections. It gained prominence during the coronavirus disease 2019 (COVID-19) pandemic as a treatment for SARS-2. Remdesivir functions as a nucleotide analog prodrug, inhibiting the viral RNA-dependent RNA polymerase (RdRp). Remdesivir has shown antiviral activity against viruses of the Filoviridae and Paramyxoviridae families, as well as the omicron strain of SARS-2 [9,10,11].

Plants synthesize a vast array of organic compounds, many of which are involved in processes such as growth, cell division, respiration, photosynthesis, reproduction, and storage. These essential compounds are known as primary metabolites. Secondary metabolites are considered promising pharmacologically active compounds because of their structural diversity and broad range of biological activities. However, their therapeutic application is often limited by poor bioavailability, instability, and potential adverse effects [12]. Nanocarrier-based delivery systems, particularly liposomes, have emerged as promising strategies for improving the pharmacokinetic properties, biological safety, and therapeutic efficacy of plant-derived compounds while preserving their biological activity.

Horse chestnut (*A. hippocastanum*) seed extracts exhibit significant antiviral effects, especially against enveloped viruses. A study by Salinas et al. (2019) [13] showed that horse chestnut extract and β-escin have virucidal and antiviral activities (concentration-dependent) against RSV (respiratory syncytial virus) in vitro in HEp-2, A549, and Vero cells. Horse chestnut extract (without β-escin) reduced weight loss, viral titers in the lungs, and inflammation in mice in vivo [13]. Peñaranda Figueredo et al. (2024) [14] investigated its effects against coronaviruses SARS-2 and CCoV (canine coronavirus): horse chestnut extract and β-escin reduced viral titers concentration-dependently without cytotoxicity. Time-of-addition tests showed inhibition during viral internalization (early stages of infection). Immunomodulatory effects included reduced nuclear factor NF-κB activation and IL-6 and TNF-α production in infected cells, suggesting a dual antiviral and anti-inflammatory role [14]. A study by Michelini et al. (2018) demonstrated virucidal, antiviral, and immunomodulatory activities against herpes simplex virus type 1 (HSV-1), vesicular stomatitis virus (VSV), and dengue virus DENV [15]. Through plaque assays and luciferase reporter assays, horse chestnut extract and β-escin inhibited viral replication and modulated cytokine production in epithelial cells and macrophages, supporting their broad-spectrum antiviral potential [13,14,15].

In previous studies, the cytotoxicity of an extract from *A. hippocastanum* against HCT-8 and MRC-5 cell lines and the antiviral activity against human coronavirus strains OC43 and 229E was determined [16,17]. In the present study, HPLC–HRMS/MS analysis was first performed to characterize the metabolite composition of the extract. A subset of the annotated constituents was subsequently selected for molecular docking based on their relative peak intensity, confidence of identification or annotation, and reported biological relevance. The docking analysis was used to explore possible molecular mechanisms contributing to the previously observed anti-coronavirus activity of the extract [16,17]. Finally, the genotoxic effect was determined with direct action of the extract and after its introduction into a carrier liposome.

## 2. Results and Discussion

### 2.1. HPLC–HRMS/MS Characterization of A. hippocastanum Seed Extract

**Figure 1:**
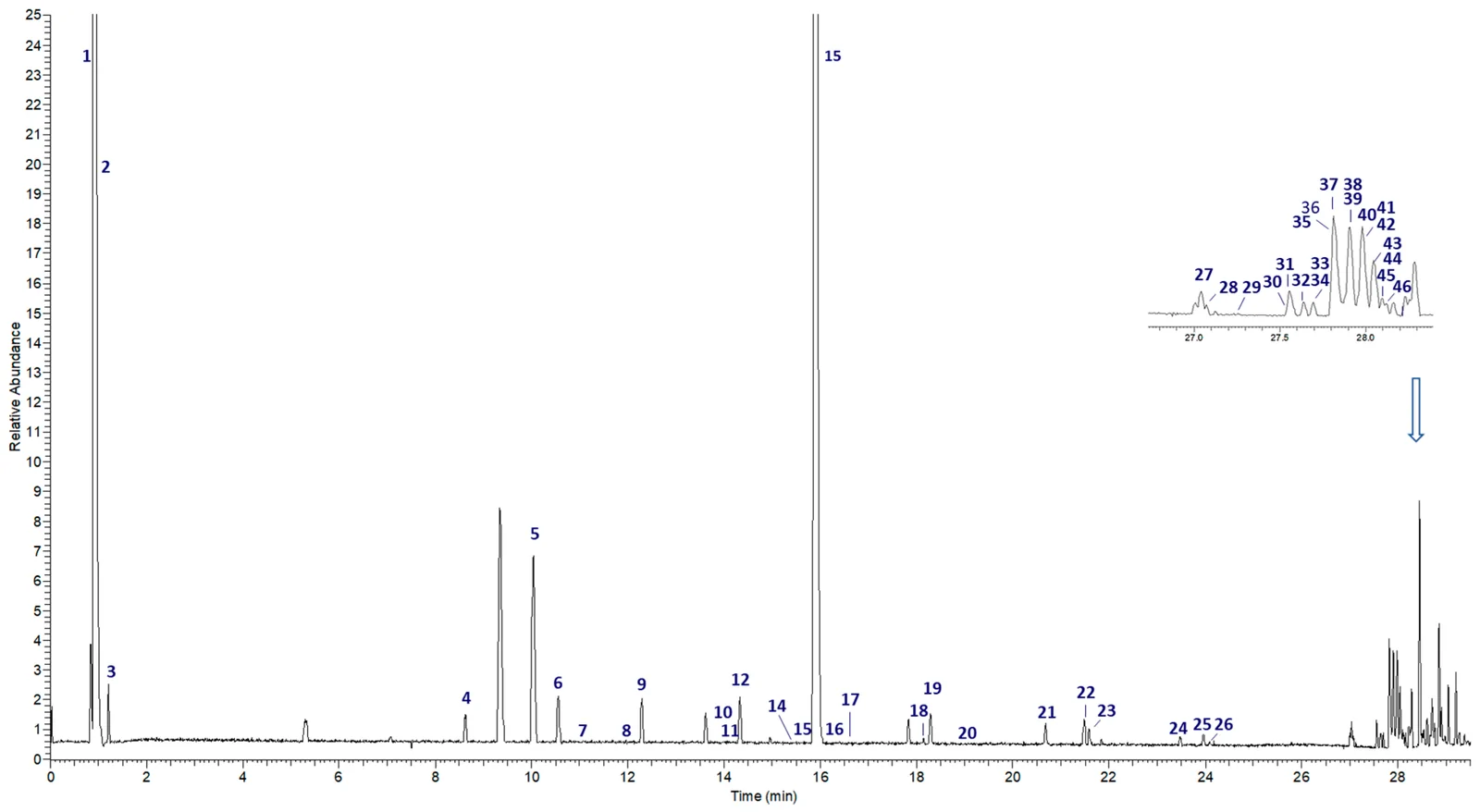
HPLC–HRMS/MS total ion chromatogram (TIC) of the *A. hippocastanum* extract acquired in negative ionization mode.

Comprehensive HPLC–HRMS/MS analysis enabled the detection and annotation of 46 metabolites in the *A. hippocastanum* seed extract (Table 1), revealing a chemically diverse profile comprising triterpenoid saponins as the largest class of annotated metabolites, together with flavonoids, indole-3-acetic acid glycosides, and low-molecular-weight organic acids. Compound annotation was achieved based on accurate mass measurements, molecular formula prediction, and characteristic MS/MS fragmentation patterns, supported by comparison with authentic standards, published data, and available spectral databases. Where authentic standards were available, compound identities were confirmed by comparison of retention times, exact mass, and MS/MS data with those of the corresponding standards. The representative total ion chromatogram (TIC) is shown in Figure 1.

The earliest eluting constituents comprised primary metabolites, including quinic (**1**), malic (**2**), and citric (**3**) acids, which were identified by comparison with authentic standards. Two indole-3-acetic acid glycosides, namely *N*-[β-d-glucopyranosyl(1→3)-[β-d-glucopyranosyl(1→4)]-β-d-xylopyranosyl]-indole-3-acetic acid (**4**) and *N*-[β-d-glucopyranosyl(1→3)]-β-d-xylopyranosyl-indole-3-acetic acid (**5**), were tentatively annotated based on HRMS/MS data. Although compounds eluting in a similar chromatographic region were previously assigned as tris-caffeoyl-spermidine and dicaffeoyl-spermidine in an LC–MS study of *A. hippocastanum* [18], the high-resolution Orbitrap data obtained in the present study support their tentative annotation as N-glycosylated indole-3-acetic acid derivatives. These annotations are consistent with the indole-3-acetic acid glycosides previously isolated and structurally characterized from *Aesculus chinensis* var. *Chekiangensis* [19], suggesting that these metabolites are also constituents of *A. hippocastanum*.

Flavonoids represented the second largest class of annotated metabolites and were dominated by glycosylated derivatives of quercetin, kaempferol, and isorhamnetin, together with several flavanone glycosides. Rutin (**8**), quercetin 3-*O*-glucoside (**10**), isorhamnetin 3-*O*-rutinoside (**13**), hesperidin (**15**), quercetin (**20**), and hesperetin (**24**) were identified by comparison with authentic standards, whereas the remaining flavonoids were tentatively assigned according to their HRMS/MS characteristics. Overall, the flavonoid profile was consistent with previous phytochemical investigations of *A. hippocastanum*, which likewise reported flavonol glycosides and flavanone derivatives as major constituents of the seed extract [18,20].

**Table 1:** Data from the HPLC–HRMS/MS analysis of *A. hippocastanum* seed extract.

Entry	Rt (min)	Compound ^a^	Molecular Formula	[M − H]^−^ (*m*/*z*)	Δppm	Fragments	Reference
1	0.94	d-(−)-Quinic acid ^b^	C_7_H_12_O_6_	191.0554	−3.66	191, 147, 111, 91, 85	
2	0.97	l-Malic acid ^b^	C_4_H_6_O_5_	133.0131	−8.65	133, 116, 115, 72, 71	
3	1.20	l-Citric acid ^b^	C_6_H_8_O_7_	191.0191	−3.48	191, 147, 121, 111, 91, 87	
4	8.60	*N*-[β-d-glucopyranosyl-(1→3)-[β-d-glucopyranosyl-(1→4)]-β-d-xylopyranosyl-indole-3-acetic acid	C_27_H_37_NO_16_	630.2057	3.81	630, 468, 262, 130	[19]
5	10.04	*N*-[β-d-glucopyranosyl-(1→3)]-β-d-xylopyranosyl-indole-3-acetic acid	C_21_H_27_NO_11_	468.1516	0.95	468, 306, 262, 174, 130	[19]
6	10.56	Quercetin *O*-dihexoside pentoside	C_32_H_38_O_21_	757.1853	2.67	757, 557, 300, 271, 255	[18]
7	11.11	Quercetin dihexoside	C_27_H_30_O_17_	625.1423	2.05	625, 463, 301, 300, 271	[18,20]
8	12.13	Rutin ^b^	C_27_H_30_O_16_	609.1466	0.82	– ^c^	
9	12.29	Kaempferol *O*-pentoside-*O*-hexoside-*O*-hexoside	C_32_H_38_O_20_	741.1903	2.60	741, 285, 284, 255, 227	[18]
10	12.45	Quercetin 3-glucoside ^b^	C_21_H_20_O_12_	463.0893	2.36	463, 301, 300, 271, 255	
11	14.11	Flavonoid glycoside I	C_27_H_30_O_16_	609.1475	2.30	609, 301, 271, 255	
12	14.33	Flavonoid glycoside II	C_27_H_32_O_14_	579.1731	2.01	579, 271, 151	
13	15.69	Isorhamnetin 3-*O*-rutinoside ^b^	C_28_H_32_O_16_	623.1631	2.13	316, 315, 301, 300, 271, 151, 107	
14	15.77	Flavonoid glycoside III	C_28_H_32_O_15_	607.1682	2.25	300, 299, 285, 284, 256, 255, 151	
15	15.90	Hesperidin ^b^	C_28_H_34_O_15_	609.1837	1.90	609, 301, 300, 286, 242, 151	
16	15.91	Flavonoid glycoside IV	C_28_H_34_O_16_	625.1775	0.16	625, 317, 299, 289, 271, 125	
17	16.68	Flavonoid glycoside V	C_27_H_32_O_14_	579.1729	1.80	271, 151	
18	18.14	Desacylescin I	C_48_H_78_O_22_	1005.4937	2.49	1005, 645, 139, 113, 101, 89, 85, 71	[21]
19	18.29	Flavonoid glycoside VI	C_28_H_34_O_15_	609.1843	2.90	609, 301, 286	[20]
20	19.02	Quercetin ^b^	C_15_H_10_O_7_	301.0360	1.90	301, 178, 151	
21	20.68	Kaempferol *O*-hexoside-*O*-rhamnoside	C_28_H_34_O_14_	593.1876	0.04	593, 285, 164, 69	[20]
22	21.48	Aesculusoside A/C	C_50_H_80_O_23_	1047.5040	2.10	1047, 867, 823, 687, 295, 179, 101, 85	[22]
23	21.59	Aesculiside M/N	C_49_H_78_O_22_	1017.4940	2.75	1017, 837, 687, 179, 101, 85	[23]
24	23.48	Hesperetin ^b^	C_16_H_14_O_6_	301.0724	2.07	301, 286, 285, 151	
25	23.96	Aesculusoside A/C	C_50_H_80_O_23_	1047.5044	2.48	1047, 867, 823, 687, 295, 179, 101, 85	[22]
26	24.09	Aesculiside M/N	C_49_H_78_O_22_	1017.4943	3.05	1017, 837, 687, 179, 101, 85	[23]
27	27.04	Escin IV/Isoescin IV	C_52_H_82_O_24_	1089.5150	2.45	1089, 909, 865, 805, 729, 179, 139, 119, 113, 101, 89, 85	[21,24]
28	27.07	Escin VII/Isoescin VII	C_51_H_80_O_23_	1059.5045	2.58	1059, 879, 729, 179, 139, 119, 113, 101, 89, 85	[21]
29	27.25	Aesculiside C/Escin XI/Isoescin XI	C_52_H_82_O_23_	1073.5155	−1.78	– ^c^	[21,23]
30	27.56	Escin VIII/Isoescin VIII	C_53_H_84_O_23_	1087.5358	2.52	1087, 907, 863, 763, 727, 179, 139, 119, 113, 101, 99, 89, 85	[21,24]
31	27.57	Deacetylescin IIa/IIb	C_52_H_82_O_22_	1057.5255	2.84	1057, 877, 727, 179, 139, 113, 101, 99, 89, 85	[24]
32	27.64	Escin VIII/Isoescin VIII	C_53_H_84_O_23_	1087.5363	2.98	– ^c^	[24]
33	27.68	Escin V/Isoescin V	C_54_H_86_O_24_	1117.5456	1.77	1117, 937, 893, 757, 179, 139, 113, 101, 89, 85	[24]
34	27.70	Aesculiside E	C_53_H_84_O_22_	1071.5406	2.29	1071, 891, 711, 639, 179, 113, 101, 99, 89	[23]
35	27.82	Escin Ia/Escin Ib/Isoescin Ia/Isoescin Ib or isomer	C_55_H_86_O_24_	1129.5457	1.84	1129, 1085, 993, 949, 905, 769, 733, 697, 645, 179, 139, 113, 101, 99, 89, 85	[24]
36	27.83	Escin IIa/Escin IIb/Isoescin IIa/Isoescin IIb or isomer	C_54_H_84_O_23_	1099.5354	2.13	1099, 919, 769, 179, 139, 113, 101, 99, 89, 85	[24]
37	27.87	Escin V/Isoescin V	C_54_H_86_O_24_	1117.5456	1.77	1117, 937, 893, 757, 179, 139, 113, 101, 89, 85	[24]
38	27.91	Escin Ia/Escin Ib/Isoescin Ia/Isoescin Ib or isomer	C_55_H_86_O_24_	1129.5453	1.48	1129, 1085, 1001, 949, 905, 805, 769, 697, 629, 179, 139, 113, 101, 99, 89, 85	[24]
39	27.92	Escin IIa/Escin IIb/Isoescin IIa/Isoescin IIb or isomer	C_54_H_84_O_23_	1099.5352	1.94	1099, 919, 769, 179, 139, 113, 101, 89, 85	[24]
40	27.96	Escin IIIa/Escin IIIb/IsoescinIIIa/Isoescin IIIb	C_55_H_86_O_23_	1113.5513	2.32	1113, 1069, 933, 889, 753, 725, 681, 611, 179, 119, 113, 101, 89, 85	[24]
41	27.99	Escin Ia/Escin Ib/Isoescin Ia/Isoescin Ib or isomer	C_55_H_86_O_24_	1129.5455	1.66	–^c^	[24]
42	28.00	Escin IIa/Escin IIb/Isoescin IIa/Isoescin IIb or isomer	C_54_H_84_O_23_	1099.5354	2.13	–^c^	[24]
43	28.04	Escin Ia/Escin Ib/Isoescin Ia/Isoescin Ib or isomer	C_55_H_86_O_24_	1129.5452	1.39	–^c^	[24]
44	28.06	Escin IIa/Escin IIb/Isoescin IIa/Isoescin IIb or isomer	C_54_H_84_O_23_	1099.5350	1.76	–^c^	[24]
45	28.10	Escin IIIa/Escin IIIb/Isoescin IIIa/Isoescin IIIb	C_55_H_86_O_23_	1113.5513	2.32	1113, 933, 889, 789, 753, 725, 681, 611, 179, 139, 119, 113, 101, 99, 89, 85	[21,24]
46	28.13	Escin VI/Isoescin VI	C_55_H_88_O_24_	1131.5618	2.23	1131, 951, 805, 771, 139, 119, 113, 101, 89, 85	[21,24]

^a^ Slash indicates that either of the listed compounds may correspond to the detected peak. ^b^ Confirmed by authentic standards. ^c^ MS/MS not acquired.

Triterpenoid saponins represented a chemically diverse class of annotated metabolites, consistent with their recognition as the characteristic phytochemical constituents of *A. hippocastanum* seeds. The detected saponins (18, 22, 23, 25–46) included desacylescin I, aesculusosides, aesculisides, deacetylescins, and numerous compounds tentatively annotated as escin/isoescin derivatives based on accurate mass measurements and comparison with published data. Because escins and isoescins are regioisomeric triterpenoid saponins that generate highly similar MS/MS fragmentation spectra, unequivocal discrimination between these compounds was not possible using the applied HPLC–HRMS/MS approach. Therefore, identical annotations assigned to multiple chromatographic peaks indicate distinct chromatographic features sharing the same tentative compound assignment rather than confirmed structural identities. Overall, the detection of acetylated and deacetylated saponins together with numerous unresolved escin/isoescin derivatives highlights the considerable structural complexity of horse chestnut saponins and is consistent with previous phytochemical studies of the species [21,22,23,24].

Collectively, the HPLC–HRMS/MS analysis demonstrates that *A. hippocastanum* seeds constitute a rich source of structurally diverse bioactive metabolites, particularly triterpenoid saponins accompanied by flavonoids and polar phenolic constituents. These findings provide the phytochemical foundation for the biological evaluation and molecular docking studies presented in the following sections.

### 2.2. Validation of the Docking Protocols

In previous studies, *A. hippocastanum* extract was found to inhibit the replication of human coronavirus strains OC43 and 229E [16,17]. In a subsequent study, the extract was incorporated into nanoliposomes, resulting in increased antiviral activity and reduced cytotoxicity at active concentrations. Consequently, the selectivity index (SI) increased from 3.7 for the free extract to 58.96 for the extract in nanoliposomes [25]. In the present study, the likely mechanism underlying the observed anti-coronavirus effect will be assessed using in silico modeling.

**Figure 2:**
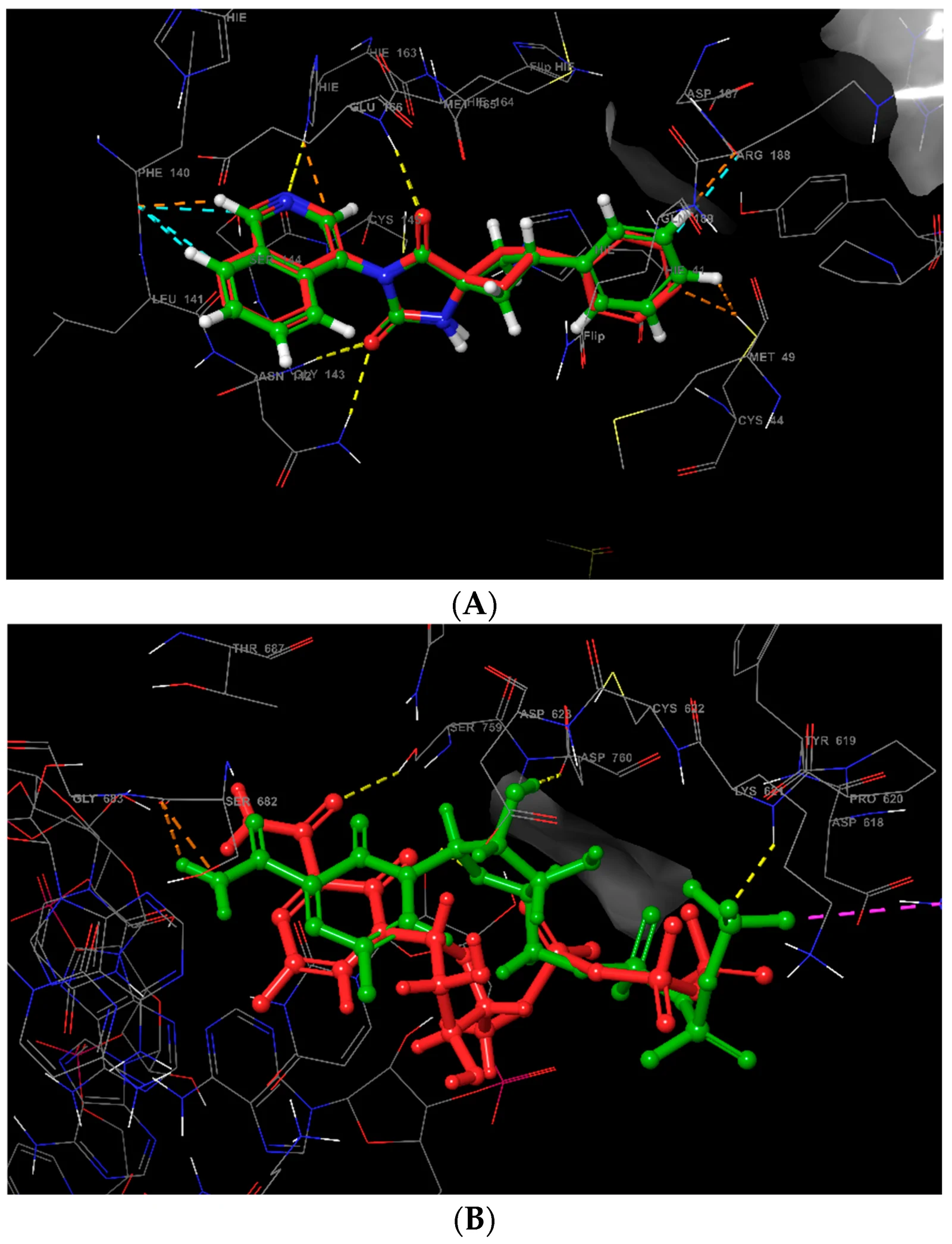
Redocked co-crystallized ligands of Nsp5 (PDB:7QBB) (Panel (**A**)) and Nsp12 (PDB:7AAP) (Panel (**B**)).

Before performing the molecular docking simulations, the docking protocols were validated through a redocking procedure. The co-crystallized ligands from the two targets, Nsp5 (PDB ID:**7QBB**) and Nsp12 (PDB ID:**7AAP**), were extracted and redocked into their respective active sites. The native ligands used for validation were the active Mpro inhibitor for 7QBB–7-isoquinolin-4-yl-2-phenyl-5,7-diazaspiro[3.4]octane-6,8-dione, and Favipiravir ribofuranosyl triphosphate for 7AAP. The redocking results showed root-mean-square deviation (RMSD) values below 2 Å, indicating that the docking protocol accurately reproduced the experimentally observed binding poses. These results confirm the reliability of the docking setup for subsequent molecular docking studies (Figure 2).

Molecular docking studies were subsequently performed using the active sites of Nsp5 and Nsp12 to prioritize phytochemicals with favorable predicted binding and to gain insights into the possible mechanisms underlying their activity. Among the 46 annotated metabolites, ten compounds were selected for docking based on (a) their relative abundance, (b) the confidence level of their annotation, and (c) their reported biological relevance, particularly as antiviral and anti-coronavirus agents. Although triterpenoid saponins represented the largest annotated chemical class, representative saponins such as escin were also evaluated in preliminary docking simulations and displayed markedly less favorable predicted binding affinities for both targets compared with flavonoids such as hesperidin and quercetin. Accordingly, flavonoids were prioritized in the main docking analysis as the most promising candidate contributors to the observed antiviral activity.

The docking scores revealed that several phytochemicals exhibit strong binding affinities, suggesting a potentially favorable interaction toward both Nsp5 and Nsp12 (Table 2).

Notably, hesperidin showed the most favorable predicted binding affinity for Nsp5 at −11.390 kcal/mol, and also a strong predicted affinity for Nsp12 at −9.241 kcal/mol. This is consistent with robust interactions that could contribute to potential dual inhibition. The stabilization of hesperidin within Nsp5 involves multiple hydrogen bonds with Thr26, Gly143, Hie163, and Hie164, which are critical residues in the active site of the protease, underscoring the compound’s stable binding [26]. Similarly, hesperidin’s binding to Nsp12 is stabilized by hydrogen bonds with Ser549 and Ser814, residues important for the polymerase function, suggesting a potentially relevant interaction from a structural perspective [27].

Comparing the docking scores of phytochemicals to that of the reference antiviral V1B (−8.16 kcal/mol for Nsp5) reveals that several natural compounds, such as hesperidin, exhibit equal or more favorable predicted binding to the synthetic inhibitor, suggesting promise for further investigation.

Quercetin also showed moderate–strong predicted binding affinities, stabilized by hydrogen bonds and π–π interactions involving residues like Glu166, Hie41, Cys44, and Asp760. Lower binding affinities observed for compounds such as citric acid and D-(−)-quinic acid suggest weaker interactions with the viral proteins, which may correlate with reduced inhibitory potential. Notably, the reference compound favipiravir ribofuranosyl triphosphate displayed strong binding only with Nsp12 (−8.90 kcal/mol), consistent with its known polymerase targeting mechanism, while lacking docking data for Nsp5.

**Figure 3:**
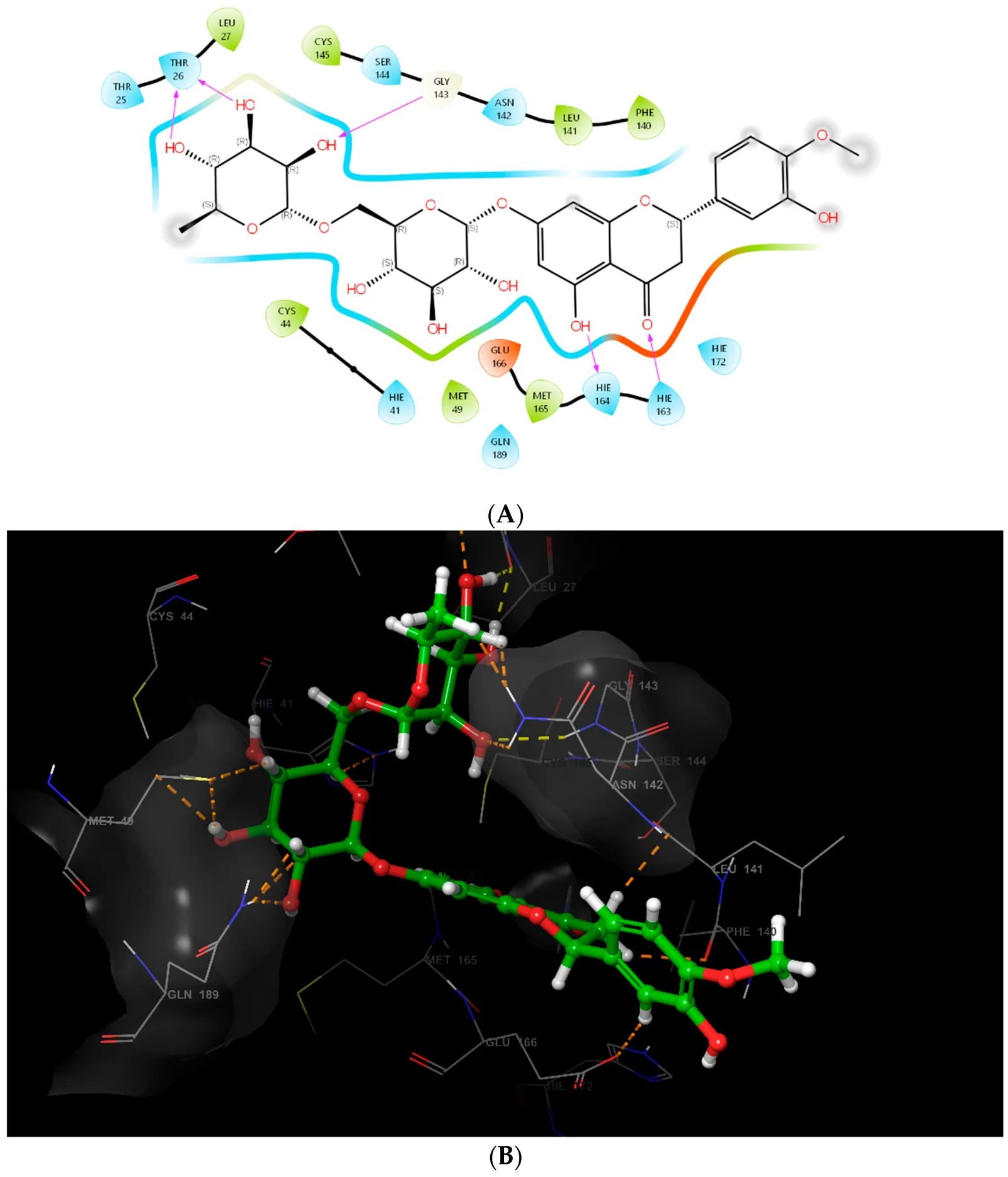
Binding conformation of hesperidin in the active site of Nsp5 (PDB ID: **7QBB**). (**A**)-2D representation; (**B**)-3D representation. The ligand is depicted in green and the hydrogen atoms in white. The hydrogen bonds are given in purple.

**Figure 4:**
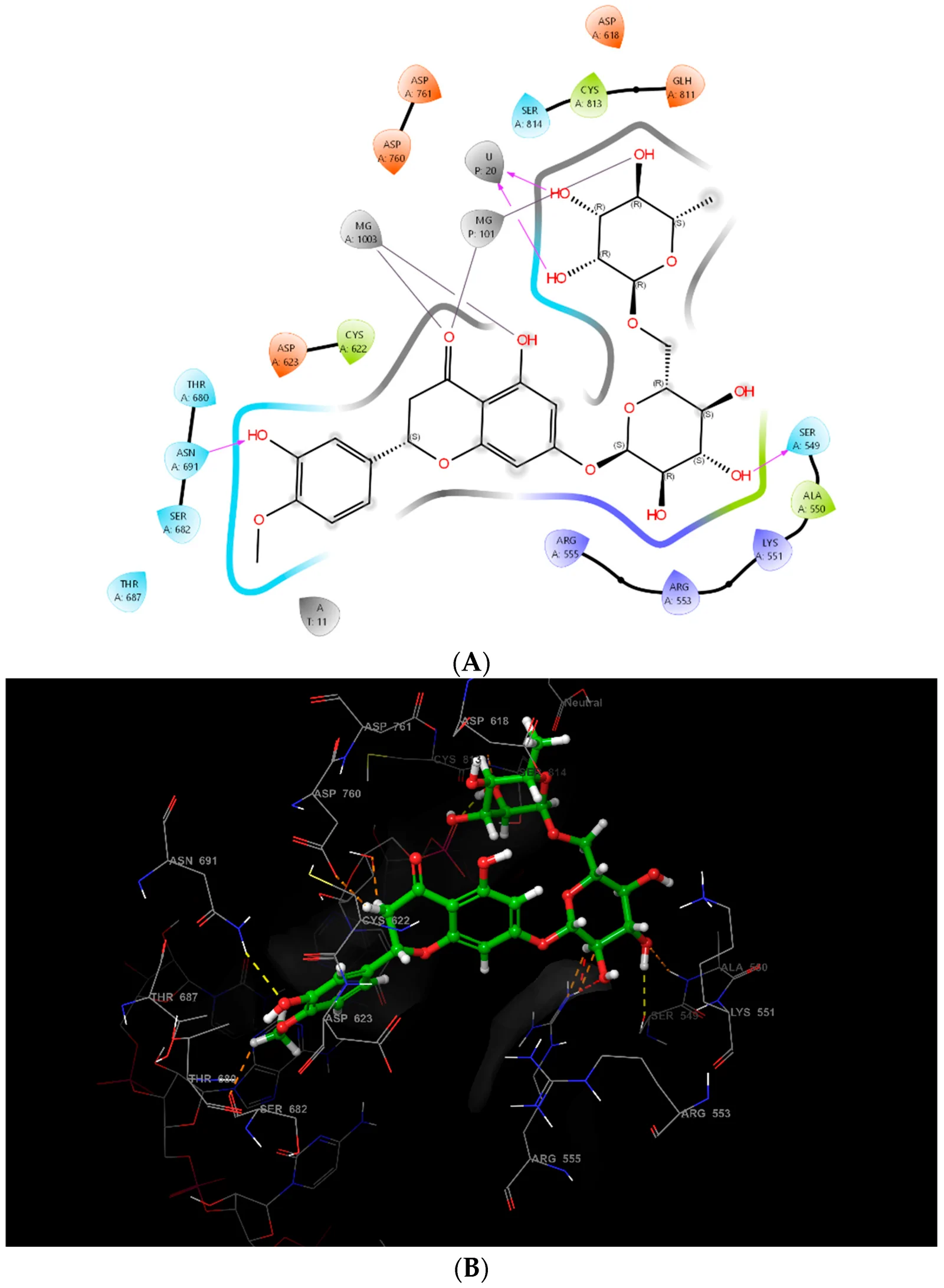
Binding conformation of hesperidin in the active site of Nsp12 (PDB:**7AAP**). (**A**)-2D representation; (**B**)-3D representation. The ligand is depicted in green and the hydrogen atoms in white. The hydrogen bonds are given in purple.

To further investigate the active amino acids involved in stabilizing hesperidin within the binding sites of Nsp5 and Nsp12, its binding conformations were analyzed in detail (Figure 3 and Figure 4).

Hesperidin was predicted to fit deeply into the substrate-binding cleft of the SARS-CoV-2 main protease (Nsp5, PDB ID:**7QBB**), adopting an extended conformation that spans the S1–S4 subsites (Figure 3). In the optimized pose, the hydrophilic sugar moiety is oriented toward the solvent-exposed entrance of the pocket, while the more hydrophobic aglycone ring system is buried in the inner part of the protein, mimicking the orientation of natural peptide substrates and known noncovalent inhibitors of Nsp5 [28]. This topography suggests that hesperidin can effectively bridge several critical recognition regions within the active site.

Moreover, multiple hydroxyl groups on the sugar and phenolic rings interact with residues such as Gly143, Ser144, and Cys145 in the S1′ region and with polar residues shaping the S1 pocket, including His163, His164, and Asn142. Beyond the catalytic region, hesperidin also occupies the S2 and S4 subsites through a combination of hydrophobic and aromatic contacts. The aglycone ring system interacts with residues such as Met49, Met165, Leu167, Gln189, and Phe140, forming van der Waals and possible π–π interactions with aromatic side chains (for example, His41 or Phe140), which are recognized as important in many structure-based designs targeting Nsp5 [29].

The docking analysis of hesperidin within the binding site of Nsp12 (PDB:7AAP) indicates a well-defined and highly stabilized binding mode that is compatible with potential inhibition of the SARS-CoV-2 RNA-dependent RNA polymerase (Figure 4). In the 3D pose, the flavanone scaffold of hesperidin is buried in the catalytic channel of Nsp12, while the sugar moiety extends along the binding groove and establishes contacts with multiple residues distributed across the finger and palm domains, suggesting a strong anchoring of the ligand within the active-site tunnel used for NTP and RNA binding [30].

A hydrogen-bonding network is also formed by the numerous phenolic and glycosidic hydroxyl groups of hesperidin with catalytically important residues located near the conserved polymerase motifs, including residues that participate in coordination of incoming NTPs and stabilization of the RNA template–primer complex. Such a pattern of interactions is consistent with previous in silico studies where ligands that engage residues in motifs A–E of Nsp12, such as Asp623, Ser682, Asn691, Asp760, and Glu811, typically exhibit favorable docking scores and are predicted to have the potential to interfere with polymerase functions [31].

In addition to polar contacts, the aromatic rings of hesperidin form multiple hydrophobic and π-related interactions with nonpolar residues in the central cavity of Nsp12, which further stabilizes the ligand. This combination of extensive hydrogen bonding and complementary hydrophobic contacts mirrors the binding characteristics described for nucleoside analogs and other small molecules targeting the RdRp active site, and supports the computational hypothesis that hesperidin can occupy a pharmacologically relevant pose that competes with natural substrates [32]. In this context, hesperidin and the other docked phytochemicals should be regarded as potential contributors to the antiviral activity of the extract, whose actual role requires experimental validation with isolated compounds. The present docking results support favorable predicted interactions but do not establish these constituents as confirmed mediators of the observed antiviral effect.

The present study has some limitations. The main one is that the study was conducted with the entire extract. Although most of the components in the extract have been identified, individual experiments with them have not yet been conducted. The second limitation is that the molecular docking was conducted only with viral non-structural proteins, with the aim of explaining the inhibitory effect on the viral replicative cycle, which became more pronounced following the incorporation of the extract into nanoliposomes. Consequently, while molecular docking identifies hesperidin and other constituents as candidate ligands with favorable predicted interactions, it does not demonstrate that any single compound is responsible for the antiviral activity observed for the whole extract. An additional limitation is the use of a single docking protocol without molecular dynamics validation, which may limit the reliability of the predicted binding modes. Subsequent studies will also pay attention to the likely impact of the identified components in the extract on key structural viral proteins important for the attachment and penetration of the virus to susceptible cells. Through these future studies, an explanation will be sought for the virucidal effect of the extract observed in the previous study, as well as its impact on the stage of viral adsorption [16,17].

### 2.3. Determination of Genotoxicity

The genotoxic potential of free and liposome-encapsulated *Aesculus hippocastanum* extract was evaluated in normal human lung fibroblasts (MRC-5, ATCC CCL-171) using the alkaline comet assay (Table 3). Cells were exposed for 24 h to the tested formulations at 1 mg/mL, a concentration selected based on previous cytotoxicity studies demonstrating a favorable biocompatibility profile, including prolonged exposure conditions of up to 120 h. Under alkaline conditions, the assay enables sensitive detection of DNA strand breaks and alkali-labile sites. DNA damage was quantified using Tail Length, %Tail DNA, Tail Moment, and Olive Tail Moment, while comet morphology was additionally evaluated using the CC0–CC3 classification.

The results obtained show that the multicomponent plant extract and liposomes with extract exhibit low genotoxic potential at concentrations of 1 mg/mL. The extract of *Aesculus hippocastanum* demonstrated a moderate genotoxic effect, but when incorporated into liposomes the genotoxic effect decreased.

**Table 3:** Quantitative comet assay parameters of the control and treated groups.

Compound Name	CTRL		H_2_O_2_		LIP		LIP-REM		AH		LAH	
	Mean	SEM	Mean	SEM	Mean	SEM	Mean	SEM	Mean	SEM	Mean	SEM
Tail length	3.800	1.356	81.167	15.474	9.743	2.007	24.222	7.205	3.571	1.360	9.111	2.611
%Tail DNA	1.901	0.610	50.395	9.621	2.536	0.986	26.002	4.877	21.301	0.368	2.984	1.523
Tail moment	0.104	0.042	67.552	16.290	1.321	0.5316	7.119	2.688	0.053	0.020	0.537	0.403
Olive Tail Moment	0.657	0.235	36.948	8.974	0.618	0.056	5.029	1.861	0.416	0.099	1.485	0.607
Classification ^a^	CC0		CC3		CC0		CC1		CC1		CC0	

^a^ CC0, no or minimal DNA damage; CC1, low DNA damage; CC2, moderate DNA damage; CC3, high DNA damage. Classification was based on comet morphology and DNA migration parameters, using untreated cells as the negative control and H_2_O_2_-treated cells as the positive control. The CC0–CC3 system was used as a semi-quantitative morphological classification and was not based on fixed numerical %Tail DNA thresholds.

The quantitative parameters of the comet assay (%Tail DNA, Tail Length, Tail Moment, and Olive Tail Moment) were calculated at the sample level, with each well/gel considered as a single sample and at least 50 randomly selected, non-overlapping comets evaluated for each sample. The mean value of the parameters for each sample was used for subsequent statistical analysis, with individual comets not being considered as independent observations, in accordance with the recommendations for comet-assay analysis. The assay response was verified using untreated cells as the negative control and H_2_O_2_-treated cells as the positive control, which exhibited minimal and pronounced DNA damage, respectively.

**Figure 5:**
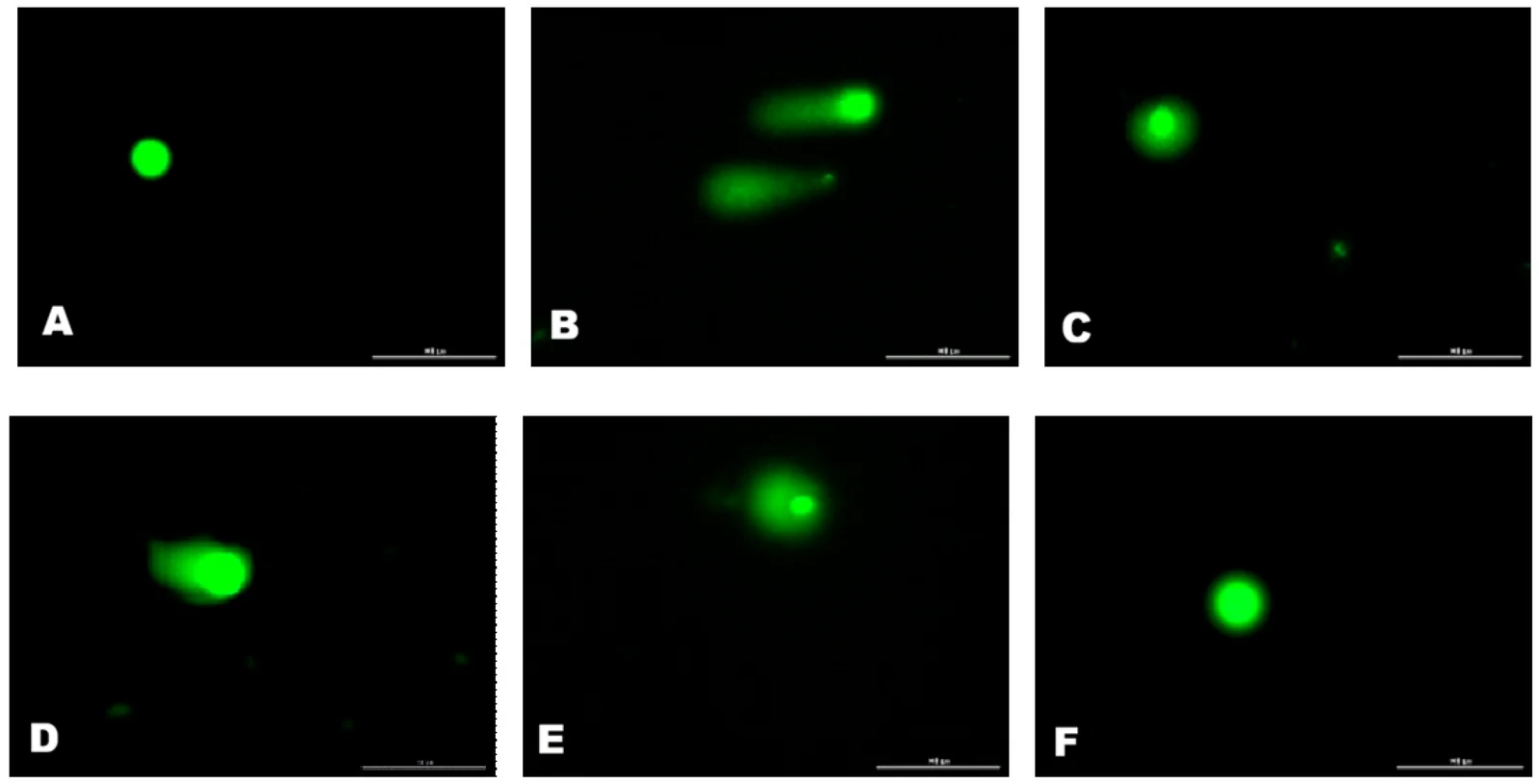
Representative comet morphologies of MRC-5 cells after 24 h treatment with the tested extracts. (**A**) Untreated control; (**B**) H_2_O_2_-treated positive control; (**C**) unloaded liposomes; (**D**) remdesivir-loaded liposomes (LIP-REM); (**E**) free *A. hippocastanum* extract (AH); and (**F**) liposome-encapsulated *A. hippocastanum* extract (LAH); the scale bar represents 100 µm.

Clear differences in DNA-damage profiles were observed among the experimental groups (Figure 5). Untreated cells exhibited a CC0 profile, whereas the H_2_O_2_-treated positive control was classified as CC3. Unloaded liposomes were classified as CC0, while LIP-REM and the free *A. hippocastanum* extract (AH) exhibited a CC1 profile; the liposome-encapsulated extract (LAH) was classified as CC0. The quantitative comet parameters were consistent with the morphological classification, supporting distinct DNA-damage profiles among the control and treatment groups. This allows for a calibrated assessment of genotoxicity and provides reliable criteria for comparison between the individual test groups.

The pure extract and liposomes with remdesivir (AH and LIP-REM) showed a CC1 profile, corresponding to low DNA damage. For the free *A. hippocastanum* extract (AH), %Tail DNA reached 21.301 ± 0.368%, whereas liposomal encapsulation markedly reduced this value to 2.984 ± 1.523% for LAH, corresponding to an approximately 86.0% reduction. The difference between AH and LAH was statistically significant (*p* < 0.01; one-way ANOVA followed by the Newman–Keuls post hoc test). It is important to note that the values are well below CC2, suggesting a potential adaptive response rather than permanent genotoxicity. This pattern is well described in the literature for polyphenols—low levels of oxidative stimuli can activate cellular defense mechanisms (Nrf2-Keap1, DNA repair pathways), leading to potential long-term biobeneficial effects [33]. The extract introduced into liposomes falls into the CC0 classification group, corresponding to no or minimal comet-assay-detectable DNA damage under the tested conditions. This classification was consistent with the quantitative comet parameters, including the lower Tail Length and Tail Moment values observed for LAH compared with the free extract. This also correlates with the absence of significant variability between different replicates.

The concordant reduction across quantitative comet parameters and morphological classifications demonstrates that, under the tested experimental conditions, liposomal encapsulation significantly attenuated DNA damage induced by the free *A. hippocastanum* extract. These findings indicate that liposomal incorporation may contribute to an improved biological safety profile of *A. hippocastanum* extract with respect to comet-assay-detectable DNA damage, supporting its further evaluation as a source of bioactive compounds for future applications.

## 3. Materials and Methods

### 3.1. Studied Extract

An aqueous-ethanolic extract of *A. hippocastanum,* produced and supplied by Extractpharma LTD, Sofia, Bulgaria, was used. The extract was prepared using a water–ethanol mixture containing 15% ethanol. The detailed method of extract preparation is presented in previous studies [16,17].

### 3.2. Nanoliposomes Studied

The procedure for formation of the liposomal carriers is described in detail in a previous study [25]. Liposomes were produced by the thin-film hydration method. Briefly, an estimated amount of lipid solution (200 µL DOPC in chloroform solution, 25 mg/mL) was dried under a stream of nitrogen by rotation to form a thin lipid layer on the flask wall. To obtain unloaded or extract-loaded liposomes, the lipid film was redispersed by adding 2 mL of water or an extract solution (10 mg/mL) to a final DOPC concentration of 2.5 mg/mL. The solution was frozen in liquid nitrogen by four consecutive freezing/heating cycles, and the stock was subjected to ultrasound for 15 min in an ice bath. In experiments, more dilute dispersions (10^−2^ mg/mL) of DOPC were prepared, followed by extrusion through a 0.20 µm filter (Minisart^®^, Sartorius, Göttingen, Germany). The liposome concentration in the samples was estimated as 3.5 × 10^14^ particles/mL.

### 3.3. HPLC–HRMS/MS Phytochemical Profiling

The phytochemical composition of the *Aesculus hippocastanum* extract was characterized by high-performance liquid chromatography coupled with high-resolution tandem mass spectrometry (HPLC–HRMS/MS) using a Vanquish UHPLC system coupled to a Q Exactive Plus^®^ hybrid quadrupole Orbitrap^®^ mass spectrometer (Thermo Fisher Scientific, Bremen, Germany), under the analytical conditions previously described in earlier studies [34,35]. Chromatographic separation was achieved on an Accucore™ C18 analytical column (150 × 2.1 mm, 2.6 μm particle size; Thermo Fisher Scientific, Bremen, Germany). The mobile phase consisted of water containing 0.1% (*v*/*v*) formic acid (solvent A) and acetonitrile (solvent B). Gradient elution was performed as follows: 0–2 min, 5% B; 2–25 min, 5–30% B; 25–30 min, 30–95% B; 30–34 min, 95% B; 34–35 min, 95–5% B; and 35–40 min, 5% B. The flow rate was maintained at 0.3 mL/min, and the injection volume was 3 μL. Prior to analysis, the extract was dissolved in a methanol/water mixture to obtain a stock solution (5000 mg/L), which was subsequently diluted with the same solvent mixture to a final concentration of 400 mg/L.

Mass spectrometric detection was performed in negative heated electrospray ionization (HESI) mode. The ion source parameters were set as follows: spray voltage, 2.90 kV; capillary temperature, 320 °C; sheath gas flow, 30 arbitrary units; auxiliary gas flow, 6 arbitrary units; sweep gas flow, 0 arbitrary units; and S-Lens RF level, 50. Nitrogen was used as both the nebulizing and collision gas. Full-scan MS spectra were acquired over an *m*/*z* range of 120–1200 at a resolving power of 70,000, using an AGC target of 1 × 10^6^ and a maximum injection time of 80 ms. MS/MS spectra were acquired using data-dependent acquisition (ddMS^2^, Top 5) at a resolving power of 17,500 with an AGC target of 1 × 10^5^, a maximum injection time of 50 ms, an isolation window of 2.0 *m*/*z*, and stepped normalized collision energies of 20, 40, and 70. Instrument control, data acquisition, and data processing were performed using Xcalibur software (version 4.2 SP1, Thermo Fisher Scientific, Waltham, MA, USA) and FreeStyle software (version 1.5, Thermo Fisher Scientific, Waltham, MA, USA). Raw HPLC–HRMS/MS data were analyzed using Compound Discoverer™ software (version 3.3, Thermo Fisher Scientific, Waltham, MA, USA) for feature detection and molecular formula prediction.

### 3.4. Chemicals and Other Consumables

The culture medium used in the experiments was Dulbecco’s modified Eagle medium (D-MEM), with dimethyl sulfoxide (DMSO) and trypsin-EDTA. Antibiotics for cell cultures (penicillin—streptomycin) were purchased from Sigma Aldrich (Darmstadt, Germany). Fetal calf serum (FBS) was supplied by Gibco-Invitrogen (Grand Island, NY, USA). The plastic vessels for cell cultures (pipettes, Petri dishes, mattresses, plates, test tubes), as well as filters (0.22 μm) for sterilizing media and solutions, were supplied by ELTA (Sofia, Bulgaria). The slides and coverslips were from Silver Quality Sigma Aldrich (Darmstadt, Germany). Ethylenediaminetetraacetic acid (EDTA), tris-hydrochloride (TRIS HCI), sodium chloride (NaCl), low-melting-point agarose (LMP), normal agarose, and all other chemicals of the highest purity available commercially were purchased from ELTA (Sofia, Bulgaria).

The reference standards for quinic acid (≥98%, CAS: 77-95-2), rutin (≥95%, CAS: 153-18-4), quercetin 3-glucoside (≥95%, CAS: 482-35-9), hesperidin (≥95%, CAS: 520-26-3), hesperetin (≥95%, CAS: 520-33-2), and quercetin (≥95%, CAS: 117-39-5) were purchased from PhytoLab GmbH & Co. KG (Vesten-bergsgreuth, Germany). LC–MS-grade solvents (acetonitrile and methanol Chromasolv^®^) were supplied by HoneywellRiedel-de Haën (Seelze, Germany). LC–MS-grade formic acid (Lichropur™, 97.5–98.5% purity, CAS: 64-18-6) was obtained from Sigma-Aldrich (Buchs, Switzerland). Ultrapure water (resistivity ≤ 0.055 μS/cm) was generated using a Smart2Pure 12 UV/UF water purification system (Thermo Electron LED GmbH, Langenselbold, Germany).

### 3.5. Cell Culture

Normal MRC-5 human lung fibroblasts (ATCC CCL-171) (American Type Culture Collection, Manassas, VA, USA) were used as a non-tumor human cell model for genotoxicity assessment. Cells were cultured in T75 tissue culture flasks in high-glucose Dulbecco’s modified Eagle medium (DMEM; Sigma-Aldrich, Taufkirchen, Germany) supplemented with 10% fetal bovine serum (FBS; Gibco, New York, NY, USA) and penicillin–streptomycin solution (Sigma-Aldrich, Germany). Cultures were maintained at 37 °C in a humidified atmosphere containing 5% CO_2_ in a Panasonic HEPA Class 100 IncuSafe MCO incubator (Nijverheidsweg, The Netherlands). Cells were routinely passaged before reaching full confluence, and only cultures in the exponential growth phase were used for the experiments. Before conducting the experiment, the cells were trypsinized with 0.25% trypsin–EDTA solution (Sigma Aldrich, Germany) for 5–7 min, the cell suspension was collected, and an aliquot was mixed with trypan blue for cell counting and viability assessment using an automated cell counter (EVE™ Automated Cell Counter, NanoEntek Europe, Martinsried, Germany). Based on the number of viable cells, the suspension was diluted with complete DMEM medium, and 1 × 10^5^ viable cells were seeded into each well of a 6-well plate (Biologix, Hallbergmoos, Germany). Twenty-four hours after seeding, the cells were exposed to the tested samples for 24 h. Each experimental condition was tested in triplicate within each experiment. Untreated cells were used as a negative control, and cells treated with 100 μM H_2_O_2_ for 30 min before performing the comet assay were used as a positive control for DNA damage.

### 3.6. Alkaline Comet Assay (Single-Cell Gel Electrophoresis)

The genotoxic potential of free *Aesculus hippocastanum* extract and its liposomal formulation was evaluated using the alkaline comet assay, also known as single-cell gel electrophoresis. This method provides high sensitivity for detecting DNA damage at the single-cell level and is a well-established tool for assessing genotoxicity induced by various chemical agents, including natural extracts.

The cells were pre-cultured in 6-well plates for 24 h in the presence of the media modified with the substances used. During the 24 h treatment period, the negative- and positive-control groups were maintained in culture medium without the tested formulations. At the end of this period, the negative-control cells remained untreated, whereas the separate positive-control group was exposed to 100 µM H_2_O_2_ for 30 min immediately before sample collection and preparation for the alkaline comet assay. H_2_O_2_ was not combined with any of the extract or liposomal treatments. For the study, 1000 µL aliquots of the cell suspension were centrifuged at 200× *g* for 5 min (on a Sigma 1–14K centrifuge, Sigma Laborzentrifugen GmbH, Osterode am Harz, Germany). The resulting cell pellet was resuspended in 500 μL of 1× PBS buffer (2.68 mM KCl, 1.47 mM KH_2_PO_4_, 1.37 mM NaCl, 8 mM Na_2_HPO_4_; pH 7) to reach a cell density of 1 × 10^5^ cells/mL. The cell suspension was mixed in a ratio of 1:10 cells with LLM agarose. The resulting microgel of cells with agarose was dropped onto glass slides, for which purpose 50 μL of the cell suspension was previously brought to a volume of 500 μL with 1× PBS buffer, then mixed with 50 μL of 1.4% low-melting agarose. The resulting 100 μL cell–agarose mixture was immediately applied onto the pre-coated slide and covered with a coverslip. The slides were incubated with cells spread on them in a cold room for 10 min at 4 °C to achieve a better-quality layer of the gel films, after which the coverslips were removed. Lysis of the cell and nuclear membranes was carried out using a lysis buffer containing 146 mM NaCl, 30 mM EDTA, pH 8, 1 mM Tris-HCl, final pH10, for 60 min at 4 °C. Electrophoresis in a buffer (0.3 M NaOH and 1 mM EDTA, pH 13) at 25 V (0.7–1.0 V/cm) and 300 mA for 20 min in the dark at 4 °C allowed chromatin loops, which the tested substances caused to breaks, to appear as comet tails, and be pulled towards the anode.

After electrophoresis, the slides were placed in neutralization buffer (0.4 M tris-HCl, pH 7.5) for 24 h at 4 °C and stained with 1× SYBR green fluorescent dye, which binds to double-stranded DNA. Comet structures were visualized and imaged using a BioTek Cytation 3 Cell Imaging Multi-Mode Reader equipped with Gen5 Image Analysis Software, version 3 (BioTek, Agilent Technologies, Winooski, VT, USA), using 4× and 20× objectives. Initial image acquisition and automated primary analysis were performed with the Gen5 software. In addition to quantitative image analysis, comet morphology was evaluated using a four-level semi-quantitative classification (CC0–CC3). Classification was based on comet head morphology and the extent and fluorescence intensity of DNA migration into the tail. Untreated cells served as the reference for normal or minimally damaged morphology, whereas H_2_O_2_-treated cells served as the positive reference for pronounced DNA damage. Accordingly, CC0 indicated no or minimal DNA damage; CC1, low DNA damage; CC2, moderate DNA damage; and CC3, high DNA damage. The categories were used for morphological assessment and were not defined by fixed %Tail DNA thresholds; quantitative DNA damage was assessed independently using Tail Length, %Tail DNA, Tail Moment, and Olive Tail Moment.

In a subsequent step, the acquired images were processed and quantitatively analyzed using ImageJ software, version 1.5 (National Institutes of Health, Bethesda, MD, USA), equipped with a comet assay analysis module. The degree of DNA damage was evaluated based on the following parameters: Tail Length, percentage of DNA in the tail (%Tail DNA), Tail Moment (%Tail DNA × Tail Length), and Olive Tail Moment.

All experiments were performed independently three times, with each treatment tested in triplicate within each experiment. Each well was processed separately for the comet assay, and at least 50 randomly selected, non-overlapping comets were evaluated per well.

### 3.7. Molecular Docking Studies

HCoV-OC43 and SARS-CoV-2 belong to the genus *Betacoronavirus* and share conserved non-structural proteins involved in viral replication, including the main protease (Nsp5, 3CLpro) and the RNA-dependent RNA polymerase (Nsp12, RdRp). The catalytic residues and overall active-site architectures of these enzymes are highly conserved across coronaviruses. High-resolution crystal structures are available for SARS-CoV-2 Nsp5 and Nsp12, whereas comparable structural data for HCoV-OC43 are limited. The three-dimensional crystal structures of the SARS-CoV-2 main protease (Nsp5; PDB ID:7QBB) [36] and the RNA-dependent RNA polymerase (Nsp12; PDB ID:7AAP) [37] were retrieved from the Protein Data Bank. Structural refinement of both proteins was carried out using Schrödinger’s Protein Preparation Wizard (Release 2025-1), which included optimization of hydrogen bond networks, assignment of appropriate protonation states, and energy minimization using the OPLS4 force field.

Molecular docking studies were conducted via the Glide protocol in the Schrödinger Maestro Suite (Release 2025-1). Docking grids were generated around the co-crystallized ligands present in each protein structure to ensure accurate binding-site definition. Ten phytoproducts were used for virtual screening against both targets. Their two-dimensional structures were drawn in Maestro’s 2D Sketcher (Release 2025-1) and subsequently converted into three-dimensional conformations. Geometry optimization, including hydrogen assignment and energy minimization, was performed using the LigPrep module with the OPLS4 force field.

### 3.8. Statistical Analysis

Processing of the photographic material was completed using BioTek Gen5 Software for Imaging & Microscopy (version 3.0), with subsequent processing using specialized software Comet Analysis–Image J, version 8.0.

The experimental data were processed using the method of variance analysis, calculating the two main statistical parameters: the arithmetic mean (x) and its error (SEM). The one-way analysis of variance (ANOVA) method was then applied. The individual groups were compared using post hoc tests (Dunnett or Newman–Keuls). In some cases, Student’s or Fisher’s *t*-tests were used for a pair of data. The differences between the groups were considered reliable at the significance level accepted for biological experiments (*p* < 0.05).

For the statistical processing of the experimental data and the construction of graphs, Microsoft Excel (Microsoft Corporation, Redmond, WA, USA, 2016) and GraphPad Prism 9.5.0 (GraphPad Software Inc., San Diego, CA, USA) were used.

## 4. Conclusions

The present study provides a comprehensive phytochemical characterization of an *A. hippocastanum* extract, while further evaluating the influence of nanoliposome encapsulation on its genotoxicity. HPLC–HRMS/MS profiling revealed a chemically diverse metabolic composition, with triterpenoid saponins representing the largest class of annotated metabolites, along with flavonoids, indole-3-acetic glycosides, and organic acids, providing the phytochemical framework for interpreting the biological findings and for selecting representative compounds for molecular docking. Genotoxicity assessment demonstrated that encapsulation of the extract into nanoliposomes reduced comet-assay-detectable DNA damage under the tested conditions, suggesting a more favorable genotoxicity profile. Molecular docking of selected extract constituents identified hesperidin as one of the phytochemicals with the highest predicted binding affinities toward the viral non-structural proteins Nsp5 and Nsp12, highlighting it as a potential contributor to the antiviral activity of the extract. However, the functional relevance of these predicted interactions requires experimental validation. Overall, these findings demonstrate that integrating phytochemical profiling with nanotechnology and computational approaches provides a rational framework for the development of *A. hippocastanum*-based antiviral formulations. Further in vitro and in vivo studies are warranted to validate the predicted molecular interactions and to establish the therapeutic potential of these formulations.

## Figures and Tables

**Table 2:** Molecular docking scores (kcal/mol) and key amino acid residues involved in stabilizing 10 phytochemicals in SARS-CoV-2 Nsp5 (PDB:**7QBB**) and Nsp12 (PDB:**7AAP**).

Compounds	Nsp5 (PDB:7QBB) kcal/mol	Key Amino Acids Involved in Stabilization	Nsp12 (PDB:7AAP) kcal/mol	Key Amino Acids Involved in Stabilization
Hesperidin	−11.390	Thr26 (two H-bonds); Gly143 (H-bond); Hie163 (H-bond); Hie164 (H-bond)	−9.241	Ser549 (H-bond); Ser814 (H-bond)
Quercetin	−8.792	Thr26 (H-bond)	−7.884	Lys545 (H-bond); Ser682 (H-bond); Asp760 (H-bond)
d-(−)-Quinic acid	−8.544	Glu166 (two H-bonds); Gln189 (H-bond)	−4.624	Asp618 (H-bond); Ser814 (H-bond)
*N*-[β-d-glucopyranosyl(1→3)]-β-d-xylopyranosyl-indole-3-acetic acid	−6.552	Asp187 (H-bond); Glu189 (H-bond)	−4.821	Lys545 (H-bond); Thr556 (H-bond)
Citric acid	−6.364	Cys44 (H-bond); Glu166 (H-bond); Asp187 (H-bond); Gln189 (H-bond)	n/a ^a^	Asp618 (H-bond); Asp761 (H-bond)
V1B ^c^	−8.16	Asn142 (H-bond); Gly143 (H-bond); Hie163 (H-bond); Glu166 (H-bond)	n.d ^b^	n.d ^b^
Favipiravir ribofuranosyl triphosphate	n.d ^b^	n.d ^b^	−8.90	Lys545 (H-bond); Asn691 (H-bond);

^a^ n/a—not available; ^b^ n.d—not determined; ^c^ 7-isoquinolin-4-yl-2-phenyl-5,7-diazaspiro[3.4]octane-6,8-dione.

## Data Availability

The raw data supporting the conclusions of this article will be made available by the authors upon request.
